# Identification of New GSK3β Inhibitors through a Consensus Machine Learning-Based Virtual Screening

**DOI:** 10.3390/ijms242417233

**Published:** 2023-12-07

**Authors:** Salvatore Galati, Miriana Di Stefano, Simone Bertini, Carlotta Granchi, Antonio Giordano, Francesca Gado, Marco Macchia, Tiziano Tuccinardi, Giulio Poli

**Affiliations:** 1 Department of Pharmacy, University of Pisa, 56126 Pisa, Italy; salvatore.galati@phd.unipi.it (S.G.); miriana.distefano@phd.unipi.it (M.D.S.); simone.bertini@unipi.it (S.B.); carlotta.granchi@unipi.it (C.G.); marco.macchia@unipi.it (M.M.); giulio.poli@unipi.it (G.P.); 2 Department of Life Sciences, University of Siena, 53100 Siena, Italy; 3 Sbarro Institute for Cancer Research and Molecular Medicine Center for Biotechnology, College of Science and Technology, Temple University, Philadelphia, PA 19122, USA; president@shro.org; 4 Department of Medical Biotechnologies, University of Siena, 53100 Siena, Italy; 5 Department of Pharmaceutical Sciences, University of Milan, 20133 Milan, Italy; francesca.gado@unimi.it

**Keywords:** virtual screening, machine learning, kinase, GSK3B

## Abstract

Glycogen synthase kinase-3 beta (GSK3β) is a serine/threonine kinase that plays key roles in glycogen metabolism, Wnt/β-catenin signaling cascade, synaptic modulation, and multiple autophagy-related signaling pathways. GSK3β is an attractive target for drug discovery since its aberrant activity is involved in the development of neurodegenerative diseases such as Alzheimer’s and Parkinson’s disease. In the present study, multiple machine learning models aimed at identifying novel GSK3β inhibitors were developed and evaluated for their predictive reliability. The most powerful models were combined in a consensus approach, which was used to screen about 2 million commercial compounds. Our consensus machine learning-based virtual screening led to the identification of compounds **G1** and **G4**, which showed inhibitory activity against GSK3β in the low-micromolar and sub-micromolar range, respectively. These results demonstrated the reliability of our virtual screening approach. Moreover, docking and molecular dynamics simulation studies were employed for predicting reliable binding modes for **G1** and **G4**, which represent two valuable starting points for future hit-to-lead and lead optimization studies.

## 1. Introduction

Glycogen synthase kinase-3 beta (GSK3β) is a serine/threonine kinase that was first characterized as a critical regulator of glycogen metabolism, specifically as an inhibitor of glycogen synthesis [1]. Over the years, it has become evident that the functional repertoire of GSK3β extends well beyond glycogen regulation, since the kinase is implicated in diverse cellular signaling pathways governing crucial biological processes. GSK3β is a ubiquitously expressed enzyme that belongs to the GSK3 family, which comprises two isoforms, GSK3α and GSK3β, each encoded by distinct genes [2]. Functionally, GSK3β operates by phosphorylating target proteins at specific serine and threonine residues, thereby modulating their activity, stability, and subcellular localization [3]. This post-translational modification gives fine control over various cellular processes, with consequences ranging from gene expression to cell proliferation and apoptosis. In recent years, the critical involvement of GSK3β in several signaling pathways of significant biological importance has been unveiled. One such pathway is the canonical Wnt/β-catenin signaling cascade, where GSK3β plays a central role in regulating the stability and nuclear translocation of β-catenin, a transcriptional coactivator [4]. Dysregulation of this pathway is associated with numerous human diseases, including various types of cancers and developmental disorders [5]. Moreover, GSK3β has been recognized as a key modulator of neuronal function and synaptic plasticity. Dysregulation of GSK3β activity has been implicated in neurodevelopmental disorders and neurodegenerative diseases, such as Alzheimer’s disease and Parkinson’s disease [6]. Furthermore, GSK3β is intricately linked to insulin signaling through its interaction with the phosphatidylinositol 3-kinase (PI3K)/Akt pathway. Akt phosphorylation of GSK3β leads to its inhibition, which, in turn, results in enhanced glycogen synthesis and cell survival [7]. Finally, GSK3β is implied in the regulation of autophagy through many different signaling pathways, which often involve the regulation of the transcriptional factor EB (TFEB) [8]. Inhibition of GSK3β has proven to stimulate autophagy in many different contexts, such as in prostate cancer cells [9], in an acute liver failure mice model [10], and an ischemia reperfusion injury rat model [11]. In particular, inhibition of GSK3β in PC12 cells promoted autophagy and decreased the aggregation and phosphorylation of α-sinuclein induced by the pesticide rotenone [12]. The role of GSK3β in multiple cellular processes and disease pathogenesis determined a significant interest in exploring its therapeutic potential. Inhibition or modulation of GSK3β activity has shown promise as a strategy to treat various disorders, including cancer and neurodegenerative diseases [13]. In particular, the use of small-molecule inhibitors of GSK3β in cancer immunotherapy may represent a valuable alternative to antibody-based immune checkpoint blockade (ICB) therapies. Specifically, the GSK3 inhibitor SB415286 proved to be as effective as the antibody-based blockade of programmed death protein 1 (PD-1) in the control of B16 melanoma or EL4 lymphoma, due to the downregulation of PD-1 obtained via GSK3 inhibition [14,15]. Moreover, the same ligand demonstrated to be more effective in suppressing B16 melanoma growth than the antibody-based blockade of lymphocyte activation gene-3 (LAG-3, which is downregulated via GSK3 inhibition), and showed to potentiate the effect of an anti-LAG-3 therapy in terms of complete clearance of tumor mass in mice, outperforming the combination of anti-LAG-3 and anti PD-1 antibodies [16]. While antibody-based ICB therapies can be limited by the insurgence of immune-related adverse events (irAEs) in patients, small-molecule inhibitors are endowed with a much more favorable pharmacokinetic profile, allowing for better distribution, tumor penetration, and response rates, which assures a higher dosage flexibility that can be properly calibrated in case of irAEs. Moreover, small-molecule inhibitors would be associated with higher patient compliance due to their possible oral administration (in contrast to the intravenous administration of antibodies) [17]. In this work, we report a machine learning-based virtual screening (VS) protocol, followed by docking evaluations and molecular dynamics (MD) simulations, carried out with the aim of discovering novel potential GSK3β inhibitors. This machine learning (ML) protocol allowed us to discover two new compounds with promising inhibitory activity against GSK3β, thus validating its predictive reliability. Furthermore, the identified inhibitors were subjected to docking studies for predicting their ligand-binding dispositions, which were further studied through MD simulations and binding free energy evaluations, thus providing a valuable starting point for hit-to-lead and future lead optimizations.

## 2. Results and Discussion

### 2.1. Development of Machine Learning Models

With the aim of generating ML models for identifying novel inhibitors of GSK3β kinase, we searched for compounds with bioactivity data related to GSK3β inhibition available on ChEMBL30 [18]. In particular, compounds with biological data expressed as IC_50_ values were collected to create the training set for building our ML models, whereas the test set for model validation was prepared using compounds with inhibitory activity measured as *K*_i_ values. After being subjected to a data curation process (see Section 3 for details), the data were classified into two categories for developing classifier ML models: active and inactive. Active compounds were defined as those with a potency ≤ 0.1 μM, while inactive compounds were those with a potency ≥ 0.5 μM. Any chemical falling within the potency range used for classification was discarded. This operation allowed the net definition of two distinct classes of compounds, as required for the proper training of binary classifiers. Although the development of multi-class predictive models trained on compounds associated with more than two possible labels (e.g., active, inactive, and borderline) may represent an interesting and valid alternative to binary classifiers, in the present work, we decided to focus our efforts on binary predictive strategies, which allowed us to obtain valuable results in both toxicological predictions [19,20] and identification of new kinase ligands trough VS [21]. Consequently, the training dataset consisted of 850 compounds classified as active and 1095 as inactive. The same classification scheme was applied to the test dataset, resulting in 209 compounds categorized as active and 429 as inactive. The t-distributed stochastic neighbor embedding (t-SNE) technique was applied to training and test compounds in order to analyze their chemical/structural composition. The results of the analysis performed with Morgan fingerprints (FPs) highlighted a high correspondence between the compounds present in both sets (Figure S1). These results confirmed the consistency of the two chemical spaces, which is ideal for the development and evaluation of ML models. A total of six different chemical FPs and the molecular descriptors calculated by the RDKit library were chosen as molecular representations to train the ML models. In particular, RDKit, Morgan, Pattern, Pharm2D, Layered, and PubChem FPs were employed. The seven different molecular representations were combined with four different ML algorithms, namely Random Forest (RF), Support Vector Machine (SVM), k-Nearest Neighbor (KNN), and Multi-layer Perceptron (MLP), resulting in 28 total ML models. A cross-validation (CV) procedure was performed on each model to obtain an indication of the model’s performance during the training phase. In conjunction with the CV, a GridSearch protocol was used to search for an ideal set of hyperparameters to optimize the models (see Section 3 for details). To focus on the global model performance, the CV results of each model were ranked according to their accuracy scores. The results shown in Figure S2 highlighted a good predictive ability of the models, which achieved average accuracy values in the range of 0.79–0.88. For the RF-based and KNN-based models, the results indicated that the type of molecular representation did not have a particularly strong impact on the model performance. On the other hand, the MLP-based and SVM-based models were more affected by the molecular representation, resulting in a more variable performance. In particular, the models generated with the RDKit descriptors yielded the lowest performance, with accuracy values around 0.80, while Morgan FPs provided higher accuracy values, with scores above 0.85. Based on the CV results, the best performing model for each algorithm used, whose accuracy score is reported in Table 1, was selected for the next validation phase. As shown in Table 1, Morgan FPs proved to be the most powerful molecular representation, producing the best predictive results in combination with each algorithm.

For each of the four selected models, a randomization test was performed to verify the hypothesis that the obtained performances were not due to chance. The analysis strongly confirmed this assumption since, as expected, accuracy values around 0.50 (representing random predictions) were obtained for each model following the randomization tests (Table S1).

Subsequently, the performance of each model was further assessed using the test set. To gain a more comprehensive understanding of the models’ predictive capabilities, precision and recall metrics were calculated in addition to the accuracy. Since, the effectiveness of a VS protocol is determined by its ability to maximize true positive predictions while minimizing false positive ones, it is important to analyze the performance of the models with more detailed metrics focused on the active predictions. In this context, precision provides a quantitative measure of the correctness of positive predictions, whereas recall quantifies the ability of the model in identifying true positives (active ligands) within the total pool of actives within the dataset (see Section 3 for details). The need for high precision is essential for ML models employed in VS studies, because the subset of compounds selected for biological evaluation is typically small compared to the initial pool of compounds within the screened library. This emphasis on precision is thus critical for optimizing hit rates in VS studies. However, it is important to note that improving the models’ ability to retain true positives while reducing false positives can lead to an increase in false negatives. This recognized challenge underscores the importance of carefully selecting the appropriate metric to evaluate the performance of the VS protocol. The results obtained from the prediction of the test set outlined a decrease in the performance of the model, which can be observed from the accuracy values reported in Table 2. However, although the overall average performance was lower than that found in the CV, the models still achieved good accuracy values. In particular, the KNN and RF models reached an accuracy of 0.69, highlighting that almost 70% of the predictions performed by these models were correct. Moreover, the two models showed a satisfying precision rate of 0.60, while the SVM and MLP models obtained values of 0.43 and 0.41, respectively. On the other hand, all models showed low recall values, which ranged between 0.16 and 0.27 (Table 2). Considering these observations, we hypothesized that the reduced overall performance of the models in terms of accuracy, compared to that found in CV, was due to an accumulation of false negative predictions. This assumption is based on the lower measured recall values, which include false negative instances, compared to the higher precision scores.

**Figure 1:**
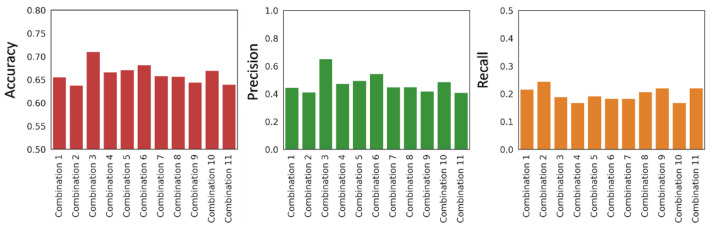
Distribution of accuracy (red bar plot), precision (green bar plot), and recall (orange bar plot) values of the eleven combinations obtained through the consensus strategy.

To improve the predictive capabilities, we applied a consensus approach, a strategy that has already shown its effectiveness in previous docking and virtual screening studies, as well as in ML-based toxicity predictions [19,20,22]. This approach consisted of combining the predictions produced by different ML models and is based on the capacity of each model to provide a probability score (PS) that is related to the confidence of the prediction. Specifically, under standard conditions, a binary classifier model provides a positive prediction if the PS is at least 0.5, otherwise it provides a negative prediction. Through the consensus approach, a new score named consensus score (CS) is calculated based on the average of the PSs provided by the combined models. Starting from the four selected models, we explored all possible model combinations to have a comprehensive overview of the performance of the models when used in combination with each other. For each of the eleven possible combinations, the CS of each compound in the test set was calculated and the new predictions were evaluated according to the same metrics used for the individual models. The analysis of the results obtained with the consensus strategy showed a distribution of performances in a range similar to that found when measuring the performances of the individual models. Nevertheless, a combination of models that produced a slight increase in the predictive ability was identified (combination 3, Figure 1). Specifically, the combination formed by the two models that obtained the best individual accuracy scores (KNN-Morgan and RF-Morgan) achieved an increase in precision (0.65) and in recall (0.19). This improvement was reflected in an increase in the accuracy score, which reached a value of 0.71 (Figure 1). These results supported the initial hypothesis of using the consensus approach, after evaluating all possible model combinations, for improving the reliability of predictions.

**Figure 2:**
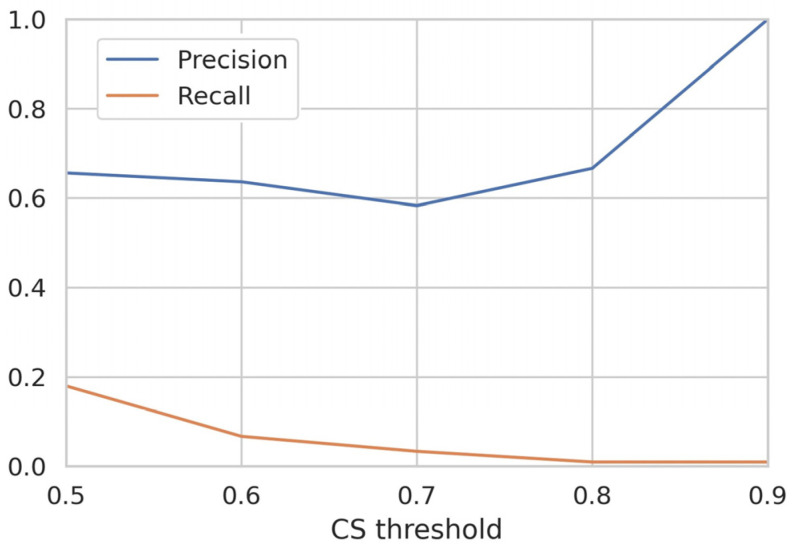
Performance evaluation obtained for the best model combination identified with the consensus approach. The precision and recall values based on test set prediction are measured using different classification thresholds.

The best consensus model was then further analyzed to observe the effect of the probability threshold on performance. Considering the goal of finding a ML-based approach suitable for VS studies, we focused on the precision score. The analysis was performed by calculating the performance of the consensus model in terms of precision using different CS thresholds. Specifically, a given compound was considered active if the CS provided by the consensus prediction was equal to or greater than the threshold used. The results obtained, as shown in Figure 2, demonstrated a stable precision value in the 0.5–0.8 range, whereas a significant increase was observed at a threshold of 0.9. As shown in Figure 2, this trend was opposite to that of recall, which decreased along with the increasing probability thresholds. This behavior can be explained by the limited number of compounds predicted to be active when using very high CS thresholds; however, this has limited relevance for VS purposes. Based on the results of the test set evaluation, the combination of the RF-Morgan and KNN-Morgan models was selected for the following VS phase.

### 2.2. Virtual Screening

Based on the results of the previous analysis, a VS study was carried out aiming at discovering novel potential inhibitors of the GSK3β enzyme by using the combination formed by the KNN-Morgan and RF-Morgan models. The sources of compounds used in this VS were the Enamine and Vitas-M commercial libraries. The compounds of the VS dataset, including a total of about 2 million molecules, were initially subjected to a data curation process similar to that applied to the training and test set compounds. Morgan FPs were then calculated for the compounds and used to generate the consensus predictions. The results of the VS showed that a total of 11,146 molecules were classified as potentially active ligands based on a CS of at least 0.5. According to the results of the analysis performed during the test set evaluation, 310 compounds with a CS of at least 0.8 were retained. A further filter was then applied to remove compounds structurally similar to molecules included in the training set. Specifically, compounds that showed a Tanimoto score of at least 0.40 with one or more molecules of the training set were removed, resulting in a total of 13 compounds that were reduced to 7 due to their prompt availability. The compounds were then subjected to a clustering procedure using RDKit FPs with a similarity cut-off of 0.70, which resulted in four clusters. For each cluster, the compound with the highest CS value was selected. The four selected molecules were then processed using MolBook UNIPI version 1.4 [23] to identify potential alerts for pan-assay interference compounds (PAINS). Since the compounds did not show any structural alert, they were purchased and subjected to biological assays to evaluate their GSK3β inhibitory activity. The results of the enzymatic inhibition assays revealed a success rate of 50%, as two of the four compounds showed promising inhibitory activity against the enzyme. In particular, compounds **G1** and **G4** presented IC_50_ values of 5810 and 640 nM, respectively (Table 3).

### 2.3. Structure-Based Studies

**Figure 3:**
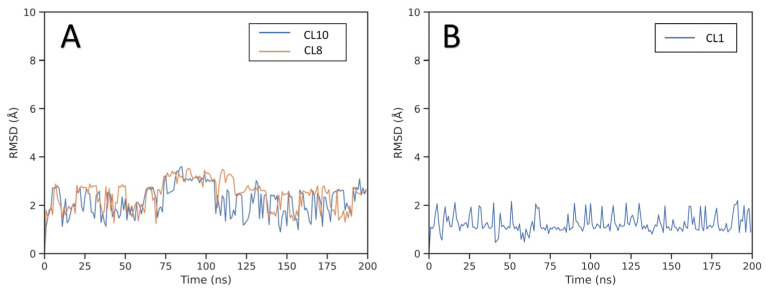
Analysis of the MD simulations of GSK3β complexed with **G1** (**A**) and **G4** (**B**).

Molecular modeling studies were performed to provide a reliable binding mode for the two active compounds identified during the VS. The first step of the structure-based protocol was carried out through docking studies to explore the possible orientations of compounds **G1** and **G4** within the binding site of the GSK3β enzyme. The docking calculations were performed with GOLD software employing the ChemScore scoring function. Due to the availability of multiple X-ray structures of GSK3β in complex with small-molecule inhibitors, the docking studies of each of the two newly identified compounds were performed using the structure of GSK3β co-crystallized with the most similar ligand available. Specifically, compound **G1** was docked into the crystal structure of GSK3β in complex with a benzofuran inhibitor (PDB code 3GB2 [24]), whereas compound **G4** was docked in the X-ray structure of GSK3β in complex with an imidazopyridine inhibitor (PDB code 4DIT [25]). The docking results obtained for each compound were then post-processed as previously performed. In particular, the obtained binding poses were clustered using a cut-off of 2.0 Å to group similar binding conformations, and the representative poses of the clusters that contained a population of at least five elements were selected. The derived poses were further filtered to retain only those that allowed the molecule to form at least one H-bond with the amino acids located in the hinge region of the protein. Based on this filtering step, two different poses (CL8 and CL10) and a single pose (CL1) were identified as putative binding conformations for compounds **G1** and **G4**, respectively. To analyze the stability of the predicted binding modes, each of the selected poses was subjected to a 200 ns MD simulation protocol. The root-mean-square deviation (RMSD) was used to evaluate the changes in the position of the ligands over the course of the simulation compared to the positions identified by the docking procedure. Figure 3B shows that the RMSD for compound **G4** is very stable with an average value of about 1.5 Å, thus supporting the stability of the proposed binding orientation.

**Figure 4:**
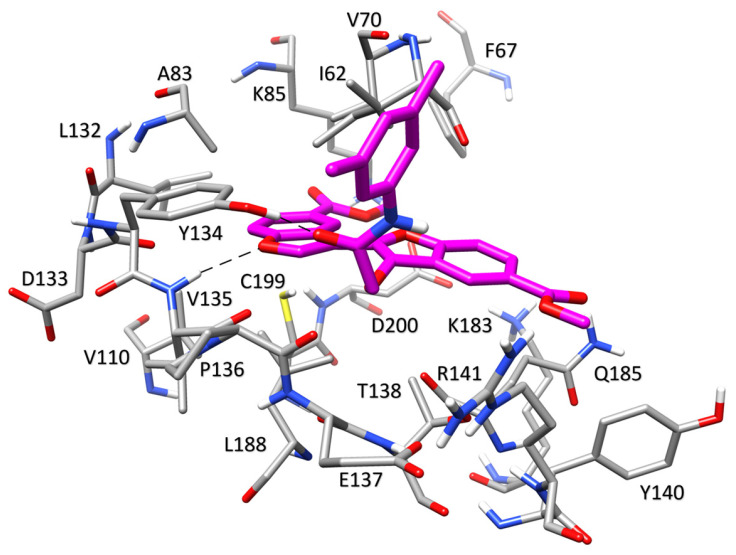
Minimized average structures of **G1** (magenta) in complex with GSK3β. The protein residues surrounding the ligand, constituting the binding site, are shown as grey sticks, whereas hydrogen bonds are shown as black dashed lines.

The RMSD results obtained from the MD simulations for both poses of compound **G1** suggest a lower stability of these binding orientations. Interestingly, through a visual inspection of the two MD simulations, we observed that the two analyzed binding orientations converged during the MD, leading to the same final binding mode. To confirm this similarity also in terms of energy, binding free energy evaluations were performed using the molecular mechanics/Poisson–Boltzmann surface area (MM-PBSA) approach [26,27,28]. This approach analyzes the MD simulation snapshots and calculates the contributions of both gas-phase and solvation free energies for the unbound ligand, unbound protein, and bound complex (see Section 3 for details). The results shown in Table S2 highlighted that the two poses achieved similar energetic values, with only a minor gap in favor of CL10, thus confirming their convergence also from an energetic point of view. Based on these results, the pose derived from cluster CL10 was selected as the representative binding mode of compound **G1**. For both compounds, the average orientation derived from the MD simulation was thus generated and appropriately refined by energy minimization to analyze the predicted binding modes. The binding mode of compound **G1**, displayed in Figure 4, shows an H-bond at the hinge region of the enzyme between the nitrogen backbone of V135 and the oxygen of the benzofuran core of the ligand. An additional H-bond formed between the amide moiety of the ligand and the hydroxyl group of Y134 contributes to increase the stability of the ligand-binding conformation. The di-substituted benzofuran core bound to the hinge region of the protein is located in a pocket delimited by V70, A83, L132, and C199, with which it forms hydrophobic contacts. Furthermore, the tri-substituted benzofuran ring shows lipophilic interactions with I62, T138, and Q185.

**Figure 5:**
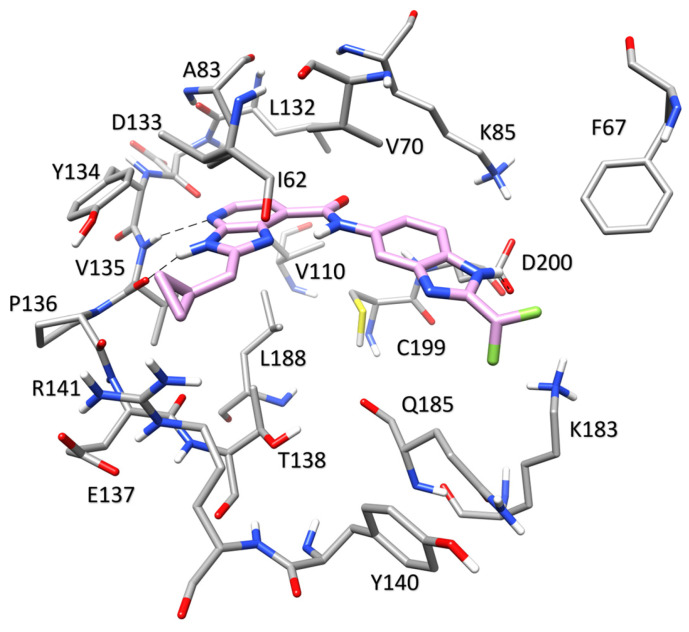
Minimized average structures of **G4** (rose) in complex with GSK3β. The protein residues surrounding the ligand, constituting the binding site, are shown as grey sticks, whereas hydrogen bonds are shown as black dashed lines.

The binding mode identified for compound **G4** is characterized by a double interaction between the imidazopyridine core of the ligand and the hinge region of GSK3β (Figure 5). The imidazole and pyridine nitrogens of the ligand form H-bonds with the backbone oxygen and nitrogen of V135, respectively.

An additional charge-assisted H-bond is also formed between the terminal benzoimidazole nitrogen of the ligand and the carboxylic group of the side chain of D200. The imidazopyridine moiety interacts with A83 on one side and L188 on the other through van der Waals interactions. The benzoimidazole ring of the ligand shows hydrophobic interactions mainly with V70, whereas the difluoromethyl substituent shows lipophilic contacts with I62, V70, K183, and Q185. Finally, the presence of an intramolecular H-bond between the imidazole ring and the amide moiety of the ligand provides additional stability to the binding mode.

To validate the structure-based protocol applied, we performed the same docking analyses, MD simulations, and binding free energy evaluations on the reference ligands of the X-ray structures considered in this work. According to the results reported in Table S3, the two poses resulting from the docking study performed on the 3GB2 complex, which converged into the same binding conformation during the MD, showed comparable binding energy values, thus confirming their convergence even from an energetic point of view. For the 4DIT complex, the pose derived from cluster CL1 showed a binding free energy gap of about 8 kcal·mol^−1^ from that calculated for the other predicted pose, thus proving to be the most energetically favored binding orientation. Figure S3 displays the overlay between the binding modes predicted for the two reference ligands and their corresponding crystallographic orientations, demonstrating the reliability of our approach in reproducing the experimental conformations of both compounds.

### 2.4. Machine Learning-Based Features Importance

**Figure 6:**
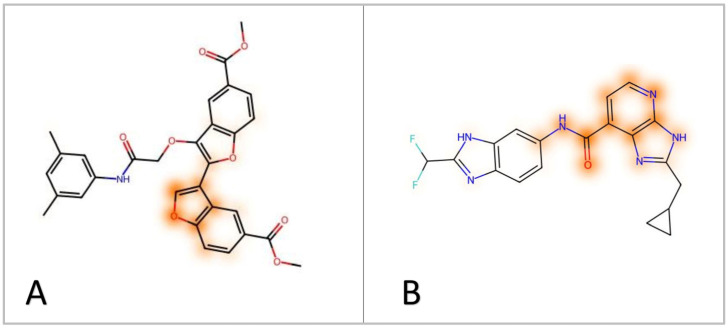
Results obtained from SHAP analysis for compounds **G1** (**A**) and **G4** (**B**). The orange colored moieties indicate a greater impact on the prediction.

Given the importance of making ML predictions explainable, we aimed at employing an automated analysis to identify the molecular features that most contributed to the prediction of activity produced for compounds **G1** and **G4** by the consensus ML learning approach used for the VS. The feature importance analysis was carried out according to the Shapley paradigm, which is a widely used approach to evaluate the impact of individual components on the final outcome derived from game theory. This method can be applied to ML models with the aim of assessing the weight of each individual feature on the final prediction. In this work, the SHAP (SHapley Additive exPlanations) approach was applied to determine the impact of individual FP bits in the generation of the consensus predictions obtained for compounds **G1** and **G4**. In particular, we applied a strategy for retro-mapping the results obtained by the Shapley method in order to highlight the structural moieties responsible for the predicted activity by employing an appropriate retro-mapping method for Morgan FP. A feature weighting method was then employed to assign a dependable score to each identified atom. In particular, the weight of the atom was given by the sum of the weights of the features in which it was contained. This weight was divided by the number of atoms in the feature and by the number of occurrences in the molecule. The final atom weight was computed as the average weight of each atom obtained from each model belonging to the best consensus approach. Finally, the mapping functions of RDKit were used to visualize the magnitude of the features that affected the prediction of the molecules (see Section 3 for details). The features importance analysis performed by using this approach for compound **G1** suggested that the di-substituted benzofuran core was the portion of the molecule with the highest impact on the prediction of activity generated by the consensus approach, thus suggesting that the ML models identified such molecular moiety as the most important for the GSK3β inhibitory activity (Figure 6A). Interestingly, the results of this analysis were in agreement with the results of our structure-based studies. In fact, based on the binding mode predicted for compound **G1** by our docking/MD simulation protocol (Figure 4), the di-substituted benzofuran core of the ligand was the structural portion of the molecule forming the key interaction with the hinge region of the protein, and thus was predicted to be fundamental for its inhibitory activity. Similarly, the results of the feature importance analysis performed for compound **G4**, highlighted the carboxamido-imidazopyridine core of the ligand as the molecular fragment that most contributed to generate the consensus prediction of activity (Figure 6B). This was consistent with the biding mode predicted for the ligand, in which the carboxamido-imidazopyridine moiety formed two H-bonds with the hinge region of the enzyme and an additional intramolecular H-bond (Figure 5), being suggested as the central scaffold at the basis of the compound’s inhibitory activity against GSK3β.

## 3. Materials and Methods

### 3.1. Modeling Datasets

Our main data source used to build the ML models was ChEMBL30 [18]. In particular, we gathered all compounds that showed inhibitory potencies against GSK3β (corresponding to UniProt ID “P49841”) measured as IC_50_. Structure refinement was carried out using the OpenEye chemistry toolkit (http://www.eyesopen.com, accessed on 2 October 2023) [29], which involved charge neutralization, removal of salts, and a structural integrity analysis. To guarantee an adequate set of compounds, for molecules with three or more available IC_50_ values, those that differed by over 25% from the respective calculated mean were eliminated. Afterward, to retain only one instance for each compound, the average IC_50_ value was recalculated and assigned as the final potency annotation. When two IC_50_ values were available for a compound, we considered the average of the two values. Following these steps, we obtained 2416 unique compounds with clearly defined activity, whose distribution is shown in Figure S4A. The distribution of the inhibitory activity of the derived molecules allowed the determination of the thresholds for the categorization of the compounds. Specifically, we classified compounds with IC_50_ values equal to or less than 0.1 µM (corresponding to pIC_50_ ≥ 7) as active, and compounds with IC_50_ values greater than 0.5 µM (corresponding to pIC_50_ ≤ 6.3) as inactive. Compounds falling in the activity range between these two thresholds were excluded from the analysis. The classification scheme resulted in a training set consisting of 850 active inhibitors and 1095 inactive compounds. An external test for model validation was prepared using compounds with inhibitory activity measured as *K*_i_. A total of 778 compounds were found in ChEMBL30 and subjected to the same data curation procedure employed for the molecules of the training set. The distribution of the *K*_i_ values of the identified compounds is shown in Figure S4B. The classification of the test set was performed with the same criteria defined for the training set, resulting in a total of 638 compounds of which 209 and 429 were labelled as active and inactive, respectively. The pipeline employed in this work is shown in Figure S5.

### 3.2. Molecular Representations

The SMILES (simplified molecular-input line-entry system) strings of training and test set compounds downloaded from ChEMBL25 were employed to compute different types of molecular FPs and molecular descriptors, in order to provide the input data for ML algorithms. In this context, we represented both molecular structures and properties using different FPs computed using RDKit software (https://www.rdkit.org, accessed on 2 October 2023) [30] and in-house python scripts. The former was used to calculate Morgan, RDKit, Layered, Pattern, and Pharm2D FPs, while the latter to compute PubChem FPs.

Morgan FPs [31], also referred to as circular FPs, are derived from Morgan’s algorithm. These FPs are utilized to depict the structure of compounds by calculating the local environment of each atom, which includes atomic bonds within a specified distance or radius. To compute these FPs, each atom identifier is transformed into a vector of fixed length using hashing functions from RDKit. In this specific study, the atomic radius was set to two, and the resulting vector length was fixed to 2048 bit.

RDKit FPs are RDKit-specific FPs. The algorithm identifies all subgraphs in the molecule within a particular range of sizes, hashes each subgraph to generate a raw bit ID, adjusts the raw bit ID to fit in the assigned FP size, and then sets the corresponding bit. In this work, a vector size of 2048 bits was used for RDKit FPs for consistency with Morgan FPs [30].

Layered FPs, also known as layered circular fingerprints, were developed to serve as substructure FPs. These FPs incorporate the same subgraph enumeration algorithm as RDKit FPs. However, instead of just generating subgraphs, they use them to set multiple bits in the FP based on various atom and bond type definitions [30].

Pattern FPs, on the other hand, were designed specifically for substructure screenings [30]. The algorithm used in Pattern FPs identifies molecular features through substructure searches using a small number of generic SMILES Arbitrary Target Specification (SMARTS) patterns. Each occurrence of a pattern is then hashed based on the atom and bond types involved, generating a FP.

Pharm2D FPs are 2D pharmacophore FPs created combining a set of chemical features with the 2D (topological) distances between them [30]. When the distances are binned, unique integer IDs can be assigned to each of these pharmacophores, and they can be stored in a FP. For this work, pharmacophore FPs were computed considering all possible combinations of default features included within the RDKit library. Specifically, the following feature types were considered: H-bond acceptor, H-bond donor, positive ionizable, negative ionizable, and aromatic features. Each feature combination included a minimum of two and a maximum of three features.

PubChem FPs are a type of substructure-based FP that are represented by a fixed-length vector of 881 bits [32]. Each bit in this vector corresponds to the presence or absence of an element or substructure in each molecule. Additionally, these FPs encode information about the count of ring systems, atom pairs, and the nearest neighbors of atoms in the molecule.

Molecular descriptors are experimentally measured or theoretically derived properties of a molecule. More specifically, they are quantitative representations of physical, chemical, or topological characteristics of molecules that summarize the molecular structure from different aspects. In this work, we employed 128 RDKit descriptors calculated with in-house python scripts.

### 3.3. Classification ML Models

Four different classification algorithms were used to develop the predictive models: Random Forest, Support Vector Machine, k-Nearest Neighbor, and Multi-layer Perceptron. The proper functions of the python library Scikit-learn [33] were used for the generation of the models.

Random Forest (RF) is an ensemble ML algorithm extensively used for both classification and regression tasks [34]. It consists of multiple individual decision trees, each trained on a bootstrap sample (sampling with replacement) from the training data. The final predictions are obtained through a majority vote of the predictions made by each individual tree. During the model building process, the main hyperparameters optimized were: (1) *max_features*, which determines the maximum number of features that can be considered when building a single tree, and (2) *n_estimators*, expressing the number of trees built before making the averages of predictions. The options of *max_features* investigated were: (a) *sqrt*, which is the square root of the total features in a single node; (b) *log2*, which corresponds to the binary logarithm of the total features for a single node; (c) *None*, for which *max_features* corresponds to the total number of features. The number of *n_estimators* that were taken into account corresponds to 100 and 500.

Support Vector Machine (SVM) maps the data according to their common patterns and aims towards their optimal division between two classes, with each of them entirely lying on opposite sides of a separating hyperplane [35]. In case of binary classification, the algorithm attempts to construct a hyperplane H by maximizing the distance between the training instances belonging to different classes. To control the magnitude of allowable training errors, the regularization hyperparameter *C* is used to balance the size of the hyperplane margins and classification errors, while the hyperparameter *kernel* is used to map the data into a higher dimensional feature space in order to make them separable. For hyperparameter *C*, the values 0.01, 0.1, 1, 10, and 100 were considered, while two types of *kernel* were tested during the tuning process: linear and Tanimoto [36].

k-Nearest Neighbors (KNN) algorithm is a type of instance-based learning, which computes the distance between the query point and the training instances to determine the k-closest points [37]. The final prediction is therefore obtained by the most frequent outcome among the features of the nearest neighbors to the input data. The hyperparameters optimized during model building were *n_neighbors* and *weight*, since they both reduce the error due to the voting of the surrounding neighbors. *n_neighbors* represents the number of neighbors taken into consideration for the classification, while *weight* indicates how much the different surrounding elements influence the prediction. In this work, two options were tested for *weight*: (a) *uniform*, indicating that all points in each neighborhood are weighted equally, and (b) *distance*, imposing that closer neighbors of a query point have a greater influence than neighbors that are further away from it. The values investigated for *n_neighbors* were in a range between 1 and 30.

Multi-layer Perceptron (MLP) is a type of feedforward artificial neural network that consists of at least three layers: an input layer, a hidden layer, and an output layer, each consisting of a set of neurons [38]. Specifically, the number of neurons in the input layer is set to the number of features for a record in the training data. The neurons contained in each hidden layer process the weighted inputs received from the neurons of the previous layer and send an output to the neurons of the following layer. Eventually, the output neurons process the inputs received from the neurons of the last hidden layer and thus provide the ultimate prediction. The main hyperparameters tuned in order to minimize the error in the path from the input to the output predictions were: (a) *hidden_layer_size*, which indicates the number of neurons and the number of hidden layers; (b) *solver*, which is important to optimize the predictions at every decision step through the different layers; (c) *activation*, which refers to the activation function and defines how the weighted sum of the input is transformed into output by one or more nodes in a network layer; and (d) *learning_rate_init*, which controls the step-size in updating the weights. In this work, three different architectures of hidden layers were tested, two of which presented three hidden layers formed by sets of [50,50,50] and [50,100,50] neurons, respectively, while the other tested architecture was formed by a single hidden layer of 100 neurons. As the type of solver, we tested *lbfgs*, which uses a limited amount of computer memory, only storing a certain number of vectors, as well as the stochastic gradients *adam* and *sgd*. Among the activation functions, we considered “*identity*”, “*logistic*”, “*tanh*”, and “*relu*” functions. The options investigated for *learning_rate_init* were 0.01, 0.001, 0.0001, and 0.00001.

### 3.4. Machine Learning Models Generation and Evaluation

Using the seven different types of molecular representations and four different ML algorithms, 28 combinations of ML models were generated. To determine the optimal hyperparameter settings, an optimization procedure using Grid Search cross-validation was applied to all generated models. Grid Search is a common method for optimizing hyperparameters in machine learning. It involves the construction of a “grid” of different hyperparameter combinations and the systematic evaluation of these combinations to identify the optimal set of hyperparameters that provides the best performance for a given model and dataset. This approach is important because hyperparameters can have a significant impact on model performance. In this work, the grid search technique was used in 5 cycles of CV, which allowed the assignment of a score calculated with the accuracy metric (vide infra). For each model, the different combinations of hyperparameters were ranked to identify the one that produced the best performance in terms of accuracy. An additional 10-fold CV with 70/30 random splitting was performed using the best hyperparameter setting. Specifically, each model was trained 10 times with a random sample corresponding to 70% of the training set, and its performance was evaluated on the remaining 30% of data. The results obtained by the different models in terms of accuracy during the 10-fold CV allowed the identification of the best performing FP for each of the four algorithms. This way, four top-scored models, one for each algorithm used, were selected.

### 3.5. Metrics

To evaluate the performance of the four top-scored models selected, we considered three key statistical parameters: precision, recall (also known as sensitivity), and accuracy [39], which are defined as follows:

$$\mathrm{Precision}=\frac{TP}{\left(TP+FP\right)}$$

$$\mathrm{Recall}=\frac{TP}{\left(TP+FN\right)}$$

$$\mathrm{Accuracy}=\frac{TP+TN}{\left(TP+TN+FP+FN\right)}$$

*TP* (true positives) and *TN* (true negatives) represent the number of correctly predicted active and inactive compounds, respectively. *FP* (false positives) is the number of inactive compounds incorrectly predicted as active, while *FN* (false negatives) is the number of active compounds incorrectly predicted as inactive. Precision measures the model’s ability to provide correct positive predictions. It calculates the ratio of correct positive predictions over the total positive predictions, which includes false positives. Recall calculates the number of correctly classified active compounds over the total number of actual active compounds. Both precision and recall yield values between 0 and 1, where 1 corresponds to the ideal model performance. Accuracy considers all values in the confusion matrix derived from binary classification. It is a global index with a range from 0 to 1, where 1 indicates perfect classification, 0.5 represents a random classification, and 0 denotes a complete inverse classification.

### 3.6. Consensus Strategy

The consensus strategy is based on the PS generated by each individual model and associated with each individual prediction. A prediction score that falls in the range 0 ≤ PS < 0.5 indicates an inactive prediction, whereas a prediction score that falls in the range 0.5 ≤ PS ≤ 1 represents an active prediction. Furthermore, the closer the score is to 1 and 0, the more reliable the prediction of activity and inactivity is, respectively. For each molecule, the consensus approach calculates the average of the PS generated by all individual models employed, which corresponds to the CS. If the CS score achieves a value of at least 0.5, the molecule is predicted to be active, while for values below 0.5, the compound is predicted to be inactive [19,20]. The statistical parameters used to evaluate the performance of each model, i.e., precision, recall, and accuracy, were also used for the assessment of the consensus approach.

### 3.7. Virtual Screening Dataset

Approximately 2 million commercial compounds derived from Enamine and Vitas-M databases were processed for structural integrity check, charge neutralization, and removal of any counter ions present, thus generating a VS dataset. The combination formed by the two models that obtained the best individual accuracy scores (KNN-Morgan and RF-Morgan) was used to predict the potential activity of the molecules contained in the VS dataset. After the prediction phase, only those instances with a CS value of at least 0.8 were retained; then, compounds that showed a Tanimoto score of at least 0.40 with one or more molecules of the training set were removed. Prior to the application of the CS of 0.8, the value of 0.9 was used; however, no compounds remained at the end of this restrictive filter procedure.

### 3.8. GSK3β Inhibition Assay

The compounds identified by the VS were purchased and their inhibitory activity against GSK3β was evaluated using the in vitro fluorescence-based Z’-LYTE assay (Thermo Fisher Scientific, Madison, WI, USA). Inhibitory potency was measured as IC_50_ values determined by logistic dose–response curves with 10 data points, each expressed as the average of two independent assays. The assessment of developmental response interference and compound fluorescence interference was performed for all tested compound concentrations. The evaluation of developmental interference included a comparison between the test compound control wells, which lacked ATP, and the 0% phosphorylation control wells (without the presence of test compound). The evaluation of test compound fluorescence interference was performed by measuring the difference between the test compound control wells that did not contain the kinase/peptide mixture (representing the null peptide control) and the 0% inhibition control. Compounds **G1** and **G4**, which showed significant inhibitory activity, did not exhibit interference reactions. Furthermore, the purchased compounds were structurally identified by 1H-NMR studies performed on a Bruker Avance III 400 MHz spectrometer.

### 3.9. Molecular Docking

The docking calculations were performed using the X-ray structure of the GSK3β enzyme in complex with literature inhibitors. In particular, compound **G1** was docked into the crystal structure of GSK3β in complex with a benzofuran inhibitor (PDB code 3GB2 [24]), whereas compound **G4** was docked in the crystal structure of GSK3β complex with an imidazopyridine inhibitor (PDB code 4DIT [25]). The ligands were built by employing the last version of the software MolBook UNIPI version 1.4 [23]. Molecular docking was performed using the GOLD 5.1 software employing the ChemScore scoring function. The residues of the binding pocket that were located within 10 Å from the center of the co-crystallized ligand were included in the docking calculations. In the docking settings, the flip ring corners option was activated, while the “allow early termination” setting was turned off. A total of 100 genetic algorithm runs were performed for each ligand, while the other settings were kept at the GOLD default values. The docking poses generated for **G1** and **G4** were grouped into clusters using an RMSD threshold of 2.0 Å. The obtained clusters were filtered considering only those that contained at least 5 poses (5% of the total solutions), and another filter was applied to retain only the dispositions in which the ligand formed at least one H-bond with the hinge region of the enzyme. The representative (top-scored) poses for each of the selected clusters were considered for further analysis.

### 3.10. Molecular Dynamics Simulations

The simulations were carried out using AMBER version 20 [40], with the ff14SB force field at a temperature of 300 K. The ligands were assigned General AMBER force field (GAFF) parameters, and their partial charges were calculated using the AM1-BCC method with the Antechamber suite of AMBER 20. The ligand–protein complexes were immersed in a rectangular parallelepiped water box, employing the TIP3P explicit solvent model, and were solvated with a 15.0 Å water cap. To neutralize the systems, either sodium or chloride ions were added to the complex. Prior to the MD simulations, we performed two stages of energy minimization. In the first stage, we applied a position restraint of 100 kcal/(mol·Å^2^) to the complex. This minimization targeted the position of water molecules and was executed through 5000 steps of steepest descent followed by conjugate gradient algorithms until a convergence of 0.05 kcal/(mol·Å^2^) was achieved. Subsequently, we subjected the entire system to energy minimization, imposing a harmonic force constant of 10 kcal/(mol·Å^2^) solely on the protein α carbons. These minimized complexes served as the initial conformations for the MD simulations. Periodic boundary conditions and particle mesh Ewald (PME) electrostatics were employed for the simulations. We initiated the MD simulation with a 0.5 ns step, using constant-volume periodic boundary conditions. During this phase, we gradually raised the temperature of the system from 0 to 300 K. Subsequently, we conducted a 3 ns equilibration stage utilizing constant-pressure periodic boundary MD. To maintain a stable system temperature, we employed the Langevin thermostat. Subsequently, we carried out additional 196.5 ns of constant-pressure MD. In total, each protein–ligand complex was processed through a 200 ns MD simulation, in which a harmonic force constant of 10 kcal/(mol·Å^2^) was applied to constrain all α carbons of the protein. The cpptraj tool version 5.1.0 from the AMBER package was used to analyze the MD trajectories.

### 3.11. Binding Energy Calculations

The results of the MD simulations obtained for the analyzed ligand–protein complexes were used as input for the evaluation of the ligand–protein binding energies, with the aim of determining the preferred binding mode of the ligands. For this analysis, we focused on the trajectories obtained in the last 200 ns of each simulation, extracting a total of 200 snapshots at 1 ns intervals. Van der Waals electrostatic and internal interactions were calculated with the SANDER module of AMBER 20, and the MOLSURF program was employed to estimate the nonpolar energies. Polar energies were calculated using the Poisson–Boltzmann methods with the MM-PBSA module of AMBER 20. We assumed dielectric constants of 1 and 80 to represent the gas and water phases, respectively.

### 3.12. Feature Contributions and Importance Mapping

The determination of contributions to model predictions was computed employing the Shapley value approach. The SHAP (SHapley Additive exPlanations) technique was initially introduced to gauge the significance of an individual participant within a cooperative team. With this method, the assessment of team members’ impact was conducted, taking into consideration their personal input to the ultimate result of a game [41]. Shapley values have demonstrated their effectiveness in providing a reliable and equitable assessment of each individual’s significance, yielding a distinct outcome defined by the subsequent principles: local precision, coherence, and zero effect. The concept underpinning the utilization of SHAP values to elucidate machine learning models is predicated on the identification of pivotal features directly linked to the model’s outcome [42]. Concentrating on binary classifiers like the models developed in this context, feature importance was furnished with a sign that corresponded to the direction of their influence. Specifically, a positive sign designated a contribution to the prediction of activity, while a negative sign corresponded to a contribution to the prediction of inactivity. Given the model-dependent nature of the SHAP approach, we decided to employ the Permutation SHAP model-agnostic method provided by the SHAP python library. This is a model-agnostic explainer that guarantees local accuracy (additivity) by iterating completely through an entire permutation of the features in both forward and reverse directions (antithetic sampling). If performed once, it produces accurate SHAP values for models with second-order interaction effects or less. Repeating this process several times with various random permutations increases the accuracy of SHAP value estimates for models with higher-order interactions. In addition, this sequential ordering scheme facilitates the reuse of model evaluations and efficiently avoids evaluating models when background values match the current input value. The Permutation SHAP method was employed to compute feature importance from the two models included in the consensus approach used for the VS. To create a coherent visualization of the outcomes derived from the SHAP analysis, the atom weights were computed utilizing a retro-mapping approach for a comprehensive assessment. In particular, an appropriate retro-mapping technique was employed for Morgan FPs, and the built-in functions of the RDKit library were used to identify the atoms responsible for each on-bit. Subsequently, a feature weighting method was implemented to assign a dependable score upon each identified atom. Specifically, the weight of each atom within a given molecule (*fw*) was computed by dividing the score of each feature containing that particular atom by the number of associated atoms (*n_Atoms_*). This value was further scaled by the frequency of the feature (*n_occ_*):

$$fw\left(a\right)=\sum_{features}\frac{fc}{n_{Atoms}n_{occ}}$$

The ultimate atom weight was determined as the average weight of each atom obtained from each model belonging to the consensus approach. Ultimately, the atom weights were depicted through the application of RDKit mapping functions.

## 4. Conclusions

The enzyme GSK3β is involved in several cellular pathways, including the Wnt/β-catenin signaling cascade and multiple autophagy-related signaling pathways, and its relevance in synaptic modulation is known, leading to its involvement in neuronal developmental disorders. The abnormal activity of GSK3β appears to be important in the development of neurodegenerative diseases such as Alzheimer’s and Parkinson’s disease, making this enzyme an attractive biological target for drug discovery. In this context, an in silico approach was developed to perform a VS for the identification of new GSK3β inhibitors. The approach involved the development of several ML models, which were then optimized and evaluated by external validation. A consensus strategy was applied to further improve the performance of the models. The best consensus combination was used to perform a VS of commercial compounds, leading to the identification of potentially active ligands. Enzyme assays revealed the inhibitory activity of two molecules, **G1** and **G4**, resulting in a hit rate of 50%. Finally, structure-based studies were performed to predict a reliable binding mode for the newly identified ligands. The results obtained confirmed the effectiveness of the in silico approach for the identification of new inhibitors of GSK3β. This strategy may thus be used in combination with receptor-based screening techniques for the development of more complex VS studies performed on larger compound database for the identification of more potent GSK3β ligands. Nevertheless, the two compounds **G1** and **G4** identified in this work, which present activities in the low/sub-micromolar range, still have the potential to be used as a starting point for future hit-to-lead and lead optimization studies for the development of novel highly active inhibitors.

## Figures and Tables

**Table 1:** Accuracy values (and corresponding standard deviation) obtained for the four selected models during cross validation.

Model	Accuracy
RF-Morgan	0.88 ± 0.02
SVM-Morgan	0.87 ± 0.01
KNN-Morgan	0.86 ± 0.01
MLP-Morgan	0.87 ± 0.01

**Table 2:** Performance scores of the four selected models obtained during the test set evaluation.

Model	Accuracy	Precision	Recall
KNN-Morgan	0.69	0.60	0.17
MLP-Morgan	0.63	0.41	0.26
SVM-Morgan	0.64	0.43	0.27
RF-Morgan	0.69	0.60	0.16

**Table 3:** Structure and GSK3β inhibitory activity of the tested compounds.

Compound ID	Structure	IC_50_ (nM)
**G1**	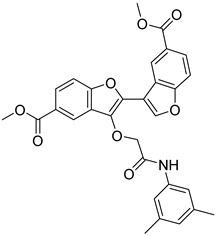	5810 ± 387
**G2**	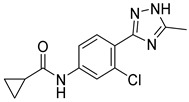	>25,000
**G3**	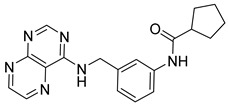	>25,000
**G4**	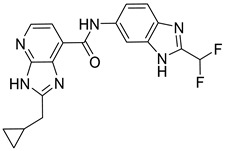	640 ± 42

## Data Availability

All compounds included in the dataset used for model building and evaluation were downloaded from ChEMBL30 (https://www.ebi.ac.uk/chembl/, accessed on 2 October 2023. The two libraries of commercial compounds screened by the model were downloaded from Enamine (https://enamine.net/, accessed on 2 October 2023) and Vitas-M laboratories (https://vitasmlab.biz/, accessed on 2 October 2023). The calculation of Morgan, RDKit, Layered, Pattern, and Pharm2D FPs was performed using the python library RDKit (https://www.rdkit.org/docs/GettingStartedInPython.html, accessed on 2 October 2023). The ML models were generated and tested employing the python library Scikit-learn (https://scikit-learn.org/, accessed on 2 October 2023). Docking calculations were performed using GOLD software (https://www.ccdc.cam.ac.uk/solutions/software/gold/, accessed on 2 October 2023). MD simulations were carried out using AMBER 20 software (https://ambermd.org/index.php, accessed on 2 October 2023).

## Supplementary Materials

The following supporting information can be downloaded at: https://www.mdpi.com/article/10.3390/ijms242417233/s1.
