# Supplementary material for: Identification of New GSK3β Inhibitors through a Consensus Machine Learning-Based Virtual Screening
Source: Int J Mol Sci. 2023 Dec 7;24(24):17233. doi: 10.3390/ijms242417233 (PMC10743990; doi:10.3390/ijms242417233)
Supplement: Supplementary file 1 [file ijms-24-17233-s001.zip › ijms-2744764-supplementary.pdf]

Supporting Information

Identification of new GSK3β inhibitors through a consensus machine learning-based virtual screening

Salvatore Galati[1,†], Miriana Di Stefano[1,2,†], Simone Bertini[1], Carlotta Granchi[1], Antonio Giordano[3,4], Francesca Gado[5], Marco Macchia[1], Tiziano Tuccinardi[1,], Giulio Poli[1]*

[1] Department of Pharmacy, University of Pisa, 56126 Pisa, Italy.

[2] Department of Life Sciences, University of Siena, 53100 Siena, Italy.

[3] Sbarro Institute for Cancer Research and Molecular Medicine, Center for Biotechnology, College of Science and Technology, Temple University, Philadelphia, PA 19122, USA

[4] Department of Medical Biotechnologies, University of Siena, 53100 Siena, Italy

[5] Department of Pharmaceutical Sciences, University of Milan, 20133 Milan, Italy.

* Correspondence: tiziano.tuccinardi@unipi.it, Tel.: +39 0502219595

† These authors equally contributed to this work.

Table of Contents

Figure S1. Overlaid projections of the training and test set. S2

Figure S2. Performance of the 28 models measured during the cross-validation. S3

Figure S3. Superposition of predicted and experimental conformations of the reference X-ray ligands. S4

Figure S4. Distribution plot of pIC_{50} and pK_i values of training and test set compounds, respectively S4

Figure S5. Pipeline employed in the protocol . S5

Table S1. Results of y-randomization test for the four selected models. S6

Table S2. MM-PBSA results for the analyzed GSK3β-**G1** complexes. S6

Table S3. MM-PBSA results for the reference complexes. S6

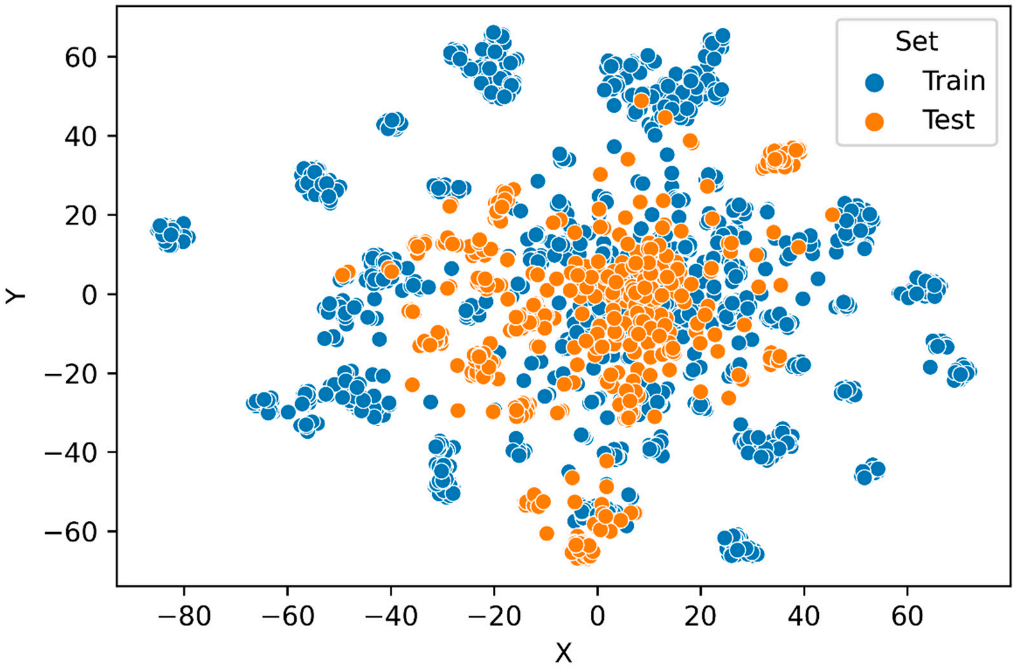

Figure S1. Overlaid projections of the training and test set. The dimensionality reduction was obtained applying the t-distributed stochastic neighbor embedding (t-SNE) algorithm on the compounds encoded as Morgan FPs.

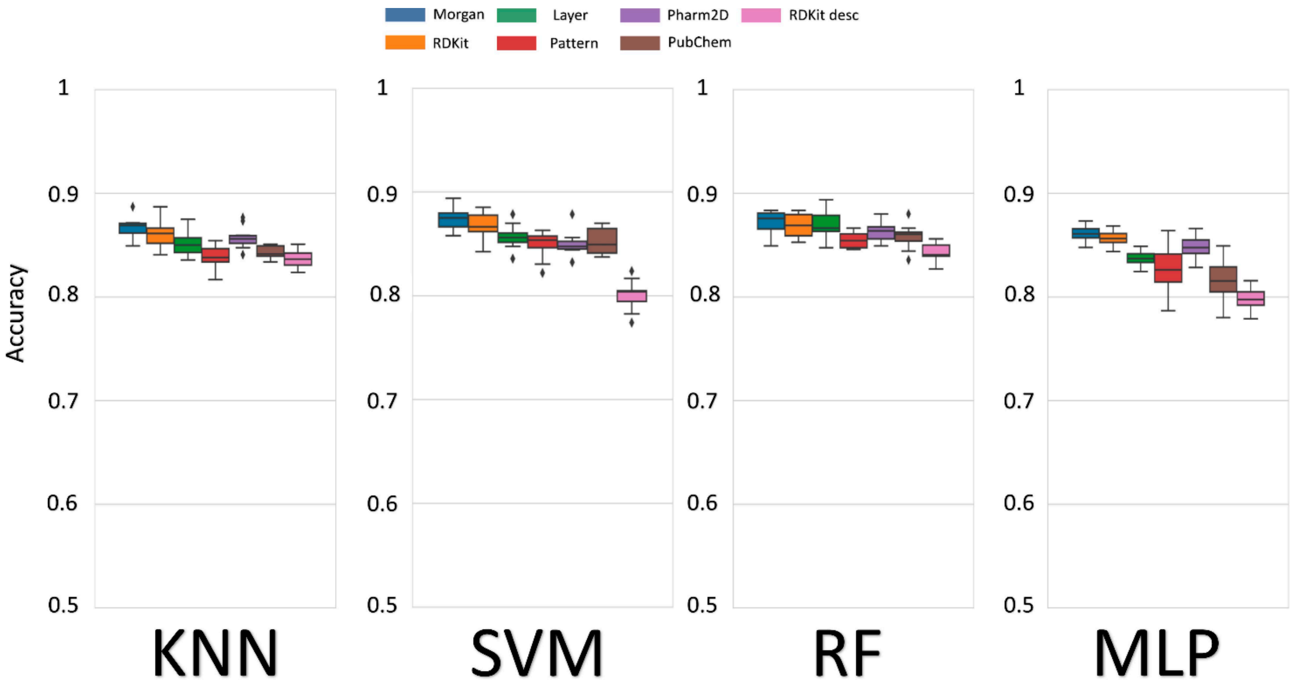

Figure S2. Performance of the 28 models measured during the cross-validation expressed in terms of Accuracy.

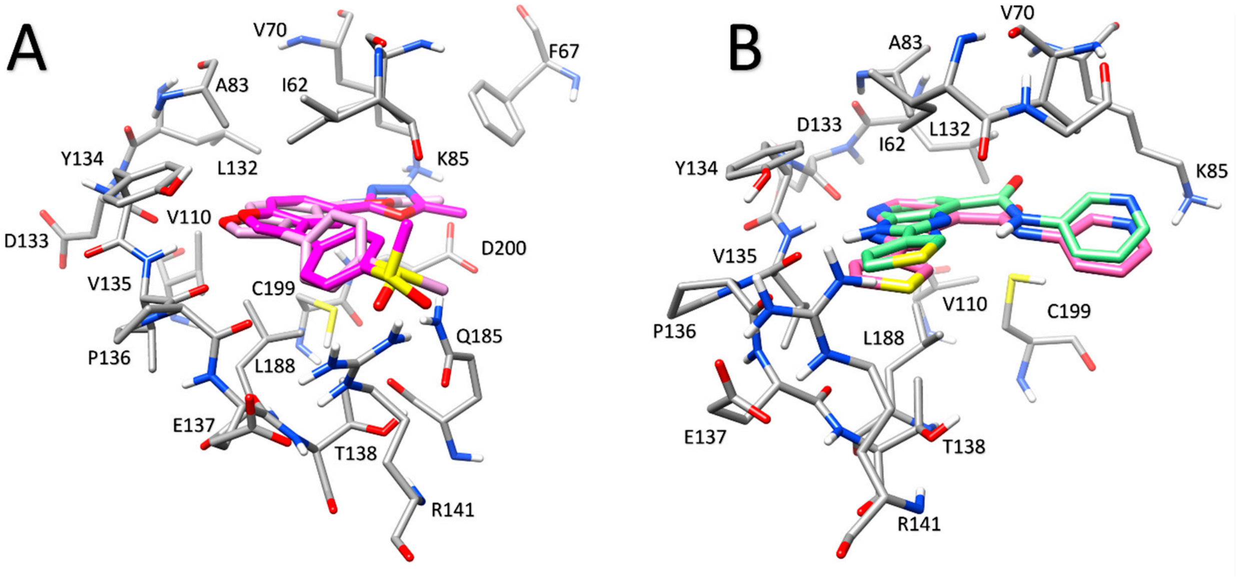

Figure S3. A) Superposition of predicted (magenta) and experimental (pink) conformations of the reference ligand of 3GB2 X-ray structure, with RMSD of 1.1 Å. B) Superposition of predicted (green) and experimental (pink) conformations of the reference ligand of 4DIT X-ray structure, with RMSD of 0.6 Å.

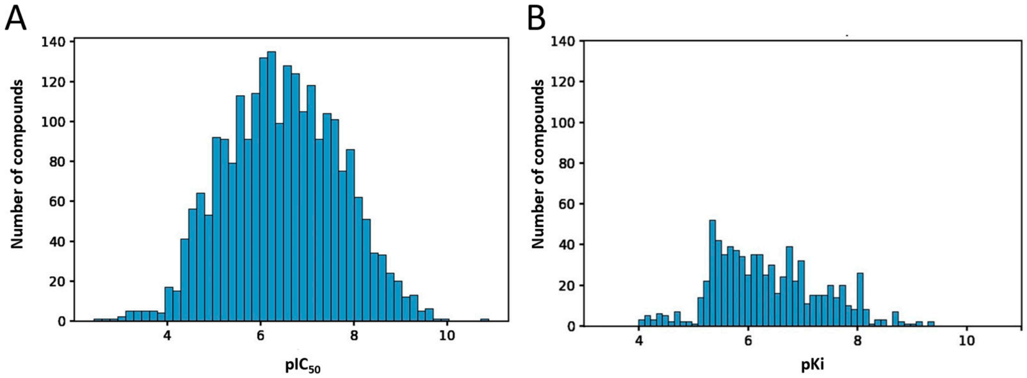

Figure S4. Distribution plot of A) pIC_{50} values of training set compounds and B) pK_i values of test set compounds.

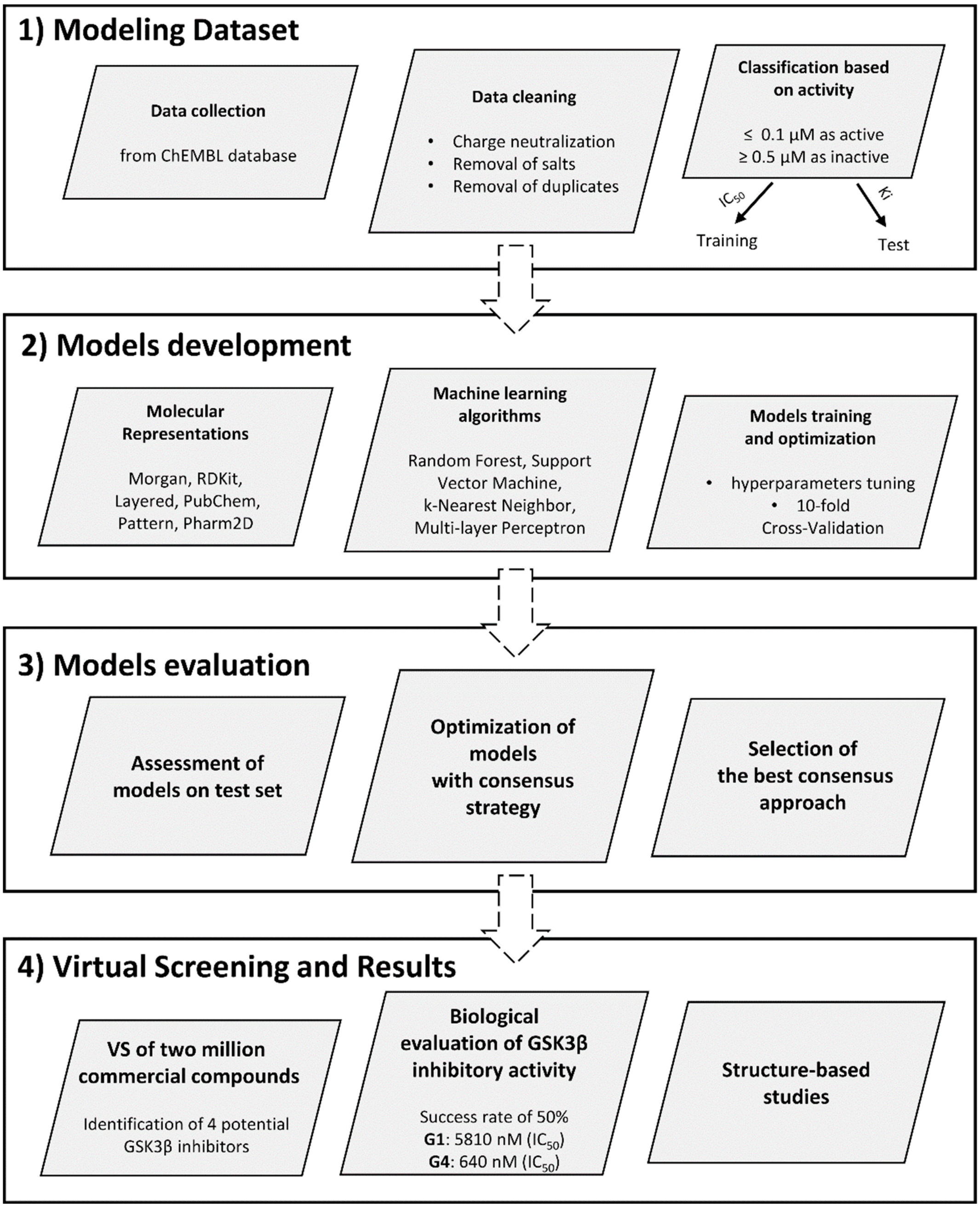

Figure S5. The pipeline used in the protocol is divided into four steps: 1) Creation of datasets to train and test models, 2) Model development and training with performance optimization, 3) Model testing and performance optimization through consensus approach, 4) Virtual screening and identification of compounds with inhibitory activity.

Table S1. Results of y-randomization test in terms of Accuracy values (and corresponding standard deviation) obtained for the four selected models.

Model	Accuracy
KNN-Morgan	0.51 ± 0.03
MLP-Morgan	0.49 ± 0.02
SVM-Morgan	0.51 ± 0.03
RF-Morgan	0.50 ± 0.01

Table S2. MM-PBSA results for the analyzed GSK3β-**G1** complexes. ΔPBSA is the sum of the van der Waals (VDW), electrostatic (ELE), as well as polar (EPB) and non-polar (ENPOLAR) solvation free energy. Data are expressed as kcal·mol^{-1}.

Pose	VDW	ELE	EPB	ENPOLAR	ΔPBSA
CL10	-50.6	-20.7	48.4	-5.0	-28.0
CL8	-51.3	-17.0	46.0	-5.0	-27.3

Table S3. MM-PBSA results for the reference complexes. ΔPBSA is the sum of the van der Waals (VDW), electrostatic (ELE), as well as polar (EPB) and non-polar (ENPOLAR) solvation free energy. Data are expressed as kcal·mol^{-1}.

		**4DIT**			
**Pose**	**VDW**	**ELE**	**EPB**	**ENPOLAR**	**ΔPBSA**
CL1	-41.2	-21.5	35.8	-3.6	-30.5
CL2	-36.0	-15.6	33.0	-3.5	-22.1
		**3GB2**			
**Pose**	**VDW**	**ELE**	**EPB**	**ENPOLAR**	**ΔPBSA**
CL1	-42.0	-24.2	43.0	-3.9	-27.2
CL2	-41.1	-25.6	43.8	-3.9	-26.8
